# Culture-based characterization of the respiratory mycobiota and antifungal resistance in bottlenose dolphins under human care

**DOI:** 10.1016/j.onehlt.2026.101439

**Published:** 2026-05-16

**Authors:** Victor Garcia-Bustos, Alba Cecilia Ruiz-Gaitán, Begoña Acosta-Hernández, Teresa Álvaro, Carlos Rojo-Solís, Mónica Valls, José Manuel Pérez-Royo, Inmaculada Rosario Medina

**Affiliations:** a University Institute of Animal Health and Food Safety (IUSA), University of Las Palmas de Gran Canaria, 35413 Arucas, Spain; b Department of Animal Pathology, Faculty of Veterinary Sciences, University of Las Palmas de Gran Canaria, 35413 Arucas, Spain; c Severe Infection Research Group, Health Research Institute La Fe, Valencia, Spain; d Oceanogràfic Valencia, Valencia, Spain

**Keywords:** *Tursiops truncatus*, Respiratory mycobiome, Antifungal resistance, Marine mammals, Azole resistance, One health

## Abstract

Fungal colonization and antifungal resistance in marine mammals remain critically understudied, despite growing recognition of their ecological and clinical importance. This study presents the first comprehensive culture-based assessment of the respiratory fungal community and antifungal susceptibility patterns in common bottlenose dolphins (*Tursiops truncatus*) maintained under human care. Using culture-based methods and differential incubation temperatures, we identified a diverse community of fungi from exhaled breath samples of ten dolphins, including clinically relevant yeasts and filamentous fungi. *Aspergillus* species were the most frequently isolated genus, with *A. niger*, *A. terreus*, and several previously unreported species in cetaceans such as *A. glaucus* and *Fusarium dimerum*. Among the yeasts, *Candida albicans*, *Nakaseomyces glabratus*, and *Meyerozyma guilliermondii* were recovered. Antifungal susceptibility testing revealed azole resistance in isolates of *Candida* and related species, *Mucor*, and *Aspergillus* spp., including multidrug-resistant profiles, while susceptibility to echinocandins and amphotericin B was preserved. Notably, resistant isolates were detected in dolphins with a history of antifungal exposure, raising concern about potential selective pressure in managed settings. No pathogenic fungi were recovered from aquarium water, suggesting endogenous or close-contact sources. These findings highlight the need for routine fungal surveillance in marine mammal facilities and contribute novel data on host-associated fungal communities in aquatic mammals. More broadly, they underscore the importance of a One Health approach to fungal ecology and resistance in marine environments.

## 1 Introduction

Emerging infectious diseases are a major global health concern, with 75% of them being zoonotic [1]. While bacteria and viruses have gathered the main attention of the scientific community, fungi have remained relatively neglected, especially in animal medicine [2]. Fungal infections are on the rise both in animals and humans [3], [4]. Recent evidence shows that they affect over 1 billion people globally, causing an estimated 11.5 million life-threatening infections and more than 1.5 million deaths annually, doubling the death toll to previously known data and surpassing tuberculosis, malaria, hepatitis, or pneumonia worldwide [4]. Climate change causes geographical spread of some fungi to traditionally non-endemic areas and is creating conditions conducive to the emergence of new fungal pathogens from previously unknown ecological niches [5].

Specifically, the marine environments harbor a diverse array of fungi, with estimates suggesting that only about 10% have been explored so far and might be a hotspot for the emergence of fungal pathogenic species that could also colonize or infect aquatic organisms [6], [7]. For instance, *Candida auris* has suddenly emerged as a new multidrug resistant fungal pathogen associated to severe outbreaks with high mortality and has been pointed out as an international threat to global public health [5]. In this regard, it has been hypothesized to emerge from marine origins under climate pressure and a potential zoonosis [8], [9]. Other new fungal emergences such as *Batrachochytrium* or *Pseudogymnascus destructants* have also been linked to climate change and have threatened the populations of amphibians and bats, respectively, towards extinction [10]. These declines can indirectly affect human health by contributing to a rise in vector-borne diseases.

In this regard, cetaceans are considered sentinel species for both ocean and human health [11]. As many marine mammals inhabit coastal regions alongside humans and share dietary sources, they can also act as reliable indicators of potential human health concerns such as zoonotic infections [12], [13]. However, they are also subject to chemical pollution and climate change, which makes them more vulnerable to infectious threats [14]. Nevertheless, while bacterial and viral threats to marine mammals have been extensively investigated, fungal pathogens remain importantly neglected, as they are in human medicine or ecosystem health [15], [16].

Emerging evidence suggests that cetaceans are susceptible to a diverse range of fungal infections caused by *Candida*, *Aspergillus*, *Cryptococcus*, and even dimorphic fungi such as *Histoplasma* spp., particularly under immunosuppressive or environmental stress conditions [17], [18], [19], [20]. Alarmingly, antifungal resistance in isolates from both wild cetaceans and individuals under human care has been documented, raising concern about their potential role as environmental reservoirs resistant strains [21], [22], [23], [24]. However, due to the difficulties in sampling wild animals, the limitations of stranded individuals due to poor conservation or contamination, and the research neglect, the ecological evidence in the mycobiome of cetaceans is remarkably limited, and only isolated reports describe antifungal susceptibility information [25].

This study aims to characterize the cultivable respiratory fungal community and to assess antifungal susceptibility patterns to clinically relevant agents in exhaled breath samples from common bottlenose dolphins (*Tursiops truncatus*) under human care.

## 2 Methods

### 2.1 Study design

This study was designed to characterize the cultivable fungal community detectable in exhaled breath of bottlenose dolphins under human care. Using a cross-sectional approach, the study captures the cultivable fungal fraction present at a defined time point and does not aim to infer long-term colonization dynamics. Non-invasive exhaled breath samples were collected from 10 dolphins housed at the Oceanogràfic aquarium in Valencia, Spain, between August and September 2023. Of the 10 dolphins included, 6 were males and 4 were females. Sampling was conducted through voluntary participation using operant conditioning to minimize stress. For each dolphin, metadata were collected including demographic characteristics, clinical history, and prior antifungal exposure; however, detailed information on treatment duration and timing was not available. Records of direct physical contact with human staff were also documented. Animal handling and sample collection procedures were conducted in accordance with the guidelines of the Animal Care and Welfare Committee of the Oceanogràfic of Valencia.

Each dolphin provided six exhaled breath samples, directly collected onto Sabouraud dextrose agar supplemented with chloramphenicol (SDA-C) (Scharlau, Barcelona, Spain), CHROMagar *Candida* medium (CHROMagar, Paris, France), and CHROMagar *Candida* supplemented with fluconazole at 32 mg/L. Inoculations were performed in duplicate, as incubation was carried out at two different temperatures, 37 °C and 22 °C. A higher temperature was used for isolation of clinically significant pathogenic fungi whilst lower temperatures were used for isolation of environmental or less thermotolerant fungi.

**Fig. 1:**
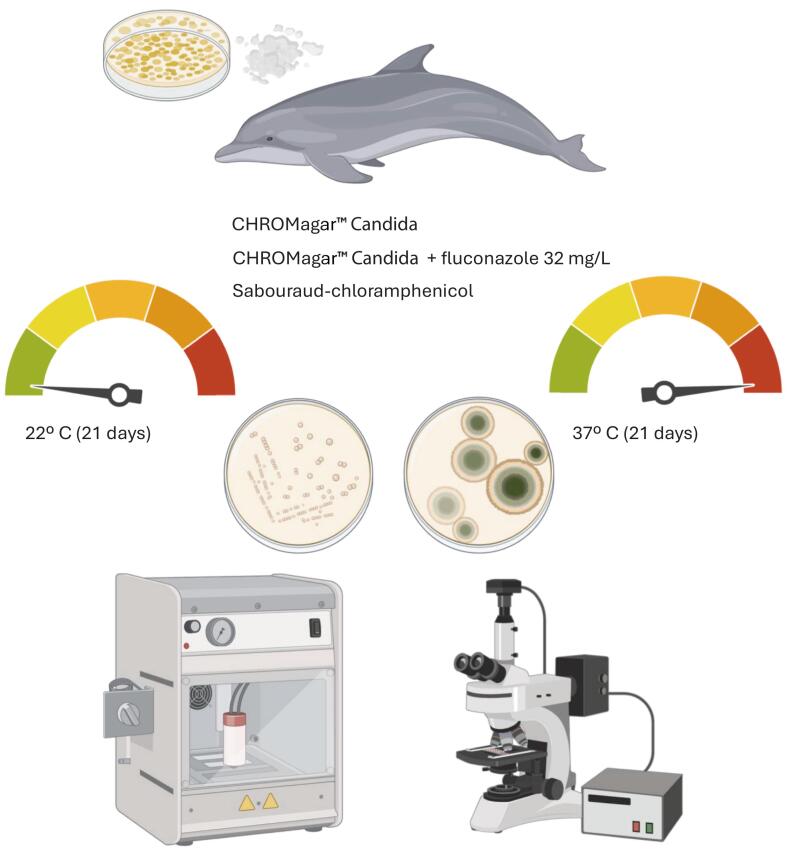
Schematic overview of the study methodology.

Samples taken on site at the aquarium were processed in less than 24 h and further studies were performed at the Health Research Institute la Fe. Cultures at both temperatures were kept in incubation for a maximum time of 21 days. All fungal growths were daily monitored, and both yeast and molds were subsequently isolated for identification. It is schematically represented in Fig. 1.

Additionally, two 5-l samples of pool water were filtered in each animal sampling day using cellulose acetate membrane filters (0.45 μm pore size, 47 mm diameter) to detect shedding of pathogenic yeast and mold species in the water environment, namely *Candida* spp., *Aspergillus* spp., *Mucorales,* and *Fusarium* spp. The filters were then directly placed onto SDA-C and incubated at 37 °C for 10 days to selectively isolate these potentially pathogenic fungi, as environmental fungi were assumed to be present. Water samples were incubated exclusively at 37 °C to selectively screen thermotolerant fungi with potential pathogenic relevance, as environmental fungi are expected to be widely distributed in aquatic systems and were not the primary target of this environmental screening.

### 2.2 Mycological study

Presumptive identification of clinically significant yeasts was performed using chromogenic media. Definitive identification was achieved by matrix-assisted laser desorption/ionization time-of-flight mass spectrometry (MALDI-TOF MS) using the MALDI Biotyper® system (Bruker Daltonics, Bremen, Germany). Filamentous fungi were identified based on macroscopic and microscopic morphology, supported by taxonomic keys. When species- or genus-level identification was not feasible through conventional methods, MALDI-TOF MS or molecular techniques were employed, including polymerase chain reaction (PCR) or sequencing of the internal transcribed spacer (ITS) region for yeasts, or β-tubulin and calmodulin genes for molds.

An exploratory screening for triazole resistance was performed in filamentous isolates with recognized pathogenic potential, specifically *Aspergillus* spp. and *Mucorales*. All isolates belonging to these genera recovered in culture were included in the screening using VIPcheck™ plates (Balis Laboratorium, Boven-Leeuwen, The Netherlands), which consist of four wells: itraconazole (4 mg/L), voriconazole (1 mg/L), posaconazole (0.5 mg/L), and one serving as a growth control without antifungal agents, respectively. Each well was inoculated with 25 μL of a fungal suspension adjusted to a 0.5 McFarland turbidity standard. Strains were classified as potentially resistant if growth was observed in any of the antifungal containing wells within 24–72 h, according to the manufacturer instructions.

Simultaneously, antifungal susceptibility study of yeasts was performed with the commercial Sensititre™ YeastOne™ YO10 AST Plates (YO10 plates) customized with isavuconazole (Thermo Fisher Scientific, Waltham, MA, USA), testing minimum inhibitory concentrations (MIC) for fluconazole, voriconazole, itraconazole, posaconazole, isavuconazole, amphotericin B, micafungin, anidulafungin, and caspofungin. When the same fungal species was recovered from a single dolphin under different culture conditions (e.g., different media or incubation temperatures), isolates were considered clonal and only one representative isolate was selected for antifungal susceptibility testing. Susceptibility was interpreted following the clinical breakpoints for fungi v. 12.0 from the European Committee on Antimicrobial Susceptibility Testing (EUCAST).

### 2.3 Statistical analysis

Statistical analyses were performed using R software (R version 4.4.1; R Foundation for Statistical Computing, Vienna, Austria). Because multiple fungal isolates could be recovered from the same dolphin under different culture conditions, analyses were conducted at the isolate level. Accordingly, these analyses were considered exploratory and aimed to describe differences in fungal recovery patterns according to culture conditions rather than infer host-level associations. Descriptive statistics were used to summarize the distribution of isolates according to culture temperature, culture medium, and morphological group. Associations between categorical variables were evaluated using Fisher's exact test or the chi-square test, as appropriate. All tests were two-tailed and a *p*-value <0.05 was considered statistically significant. To further describe fungal diversity across individuals and culture conditions, species richness (number of distinct fungal species) was calculated per dolphin and per culture temperature. Additionally, a species accumulation curve based on random permutations of dolphin sampling order was generated to assess the relationship between sampling effort and cumulative fungal species detected.

### 2.4 Ethical statement

This study involved non-invasive sampling of animals under human care and did not include experimental manipulation or procedures causing pain, suffering, or distress. All activities were conducted in compliance with applicable institutional and international guidelines for animal welfare. The study adhered, where applicable, to the ARRIVE (Animal Research: Reporting of In Vivo Experiments) guidelines and was conducted in accordance with the European Directive 2010/63/EU on the protection of animals used for scientific purposes. The study protocol was developed collaboratively by the Health Research Institute La Fe, the University Institute of Animal Health and Food Safety (IUSA) at the University of Las Palmas de Gran Canaria, and Fundació Oceanogràfic, and was approved by the Animal Ethics Committee of Fundació Oceanogràfic. Both male and female animals were included in the study; however, the influence of sex on the results was not specifically assessed due to the exploratory nature and limited sample size.

## 3 Results

Of the total dolphins tested, 9 had received prior antifungal treatment, and 8 had a documented history of previous suspected or diagnosed fungal infection. Itraconazole was administered orally to 8 dolphins, with 7 receiving it as part of a therapeutic regimen and 1, without a history of documented or suspected fungal infection, received it as long-term prophylaxis due to immunosuppression. Nystatin was given orally to 7 dolphins, and terbinafine was administered either orally to 4 dolphins or via nebulization in 1 dolphin. Fluconazole was used in 3 dolphins, while voriconazole was used in 1. One dolphin received only nystatin during the treatment period. This treatment history is reflected in Table 1. Specific administration dates were not available. All individuals had regular direct physical contact with training and veterinary staff.

**Table 1:** Characteristics of sampled dolphins. PO: per os; oral therapy.

ID	Year of birth	Sampling Date	Sex	Previous fungal Infection	Date of previous infection	Previous antifungal treatment	Type of previous antifungal
D1	2013	2023-08-09	Male	Yes	2022–06	Yes	Itraconazole PO, Nystatin PO
D2	1990	2023-08-09	Female	No	2009–01	Yes	Itraconazole PO
D3	1990	2023-08-08	Male	Yes	2023–05	Yes	Itraconazole PO, Terbinafine PO, Nebulized terbinafine, Voriconazole PO, Fluconazole PO
D4	2020	2023-08-08	Male	No		No	
D5	2006	2023-09-22	Male	Yes	2023–09	Yes	Itraconazole PO, Fluconazole PO, Terbinafine PO, Nystatin PO
D6	1995	2023-09-22	Male	Yes	2022–03	Yes	Itraconazole PO, Terbinafine PO, Nystatin PO, Fluconazole PO
D7	2003	2023-09-22	Female	Yes	2020–03	Yes	Itraconazole PO, Nystatin PO
D8	1990	2023-09-22	Female	Yes	2022–02	Yes	Itraconazole PO, Terbinafine PO, Nystatin PO
D9	2014	2023-08-08	Male	Yes	2021–11	Yes	Itraconazole PO, Nystatin PO
D10	2017	2023-09-22	Female	Yes	2022–03	Yes	Nystatin PO

Overall, 19 fungal taxa were identified across the study population. A total of 91 fungal isolates were observed: 21 yeasts (23.08%) and 70 (76.92%) filamentous fungi. This relates to a mean of 9.1 isolates per individual, with a minimum of 4 isolates in one individual to a maximum of 17 isolates in another dolphin. Among these, 54 isolates (59.34%) grew at 22 °C, while 37 isolates (40.66%) grew at 37 °C. The number of fungal species detected per dolphin ranged from 2 to 11, with a median of 4.5 species per individual. The highest species richness was observed in dolphin D7 (11 species), whereas the lowest was detected in dolphin D8 (2 species). A summary of isolate counts and species richness per dolphin is provided in Supplementary Table S1 and Supplementary Fig. S1. Species richness varied across dolphins (Supplementary Table S1). The species accumulation curve showed a rapid increase in detected taxa with the first sampled individuals, followed by a gradual leveling-off as additional dolphins were included (Supplementary Fig. S3). Although the curve approached saturation, a clear asymptote was not reached, suggesting that additional sampling could potentially reveal further fungal taxa within this population.

Culture temperature significantly influenced the isolation of fungal species from the respiratory samples of bottlenose dolphins (Fisher *p* = 0.04). At 22 °C, there was a higher prevalence of filamentous fungi (46 isolates) compared to 37 °C (24 isolates). Conversely, yeast isolates were more frequent at 37 °C (13 isolates) than at 22 °C (8 isolates). Species richness was also higher at 22 °C than at 37 °C (Supplementary Fig. S2).

A total of 39 isolates (42.86%) grew in CHROMagar, 30 isolates (32.96%) in CHROMagar with 32 mg/L fluconazole, and 22 isolates (24.17%) in SDA-C. No significant differences were observed in the prevalence of yeasts or filamentous fungi isolation according to the used medium (Fisher *p* = 0.232). The isolation efficiency of filamentous fungi in relation to yeasts per medium was highest in CHROMagar with fluconazole supplementation (86.7%), followed by SDA-C (77.3%) and CHROMagar (69.2%), whereas yeast recovery was highest in CHROMagar (30.8%), decreasing in SDA-C (22.7%) and CHROMagar with fluconazole supplementation (13.3%). A detailed characterization of the isolates per animal is presented in Supplementary table S2.

**Fig. 2:**
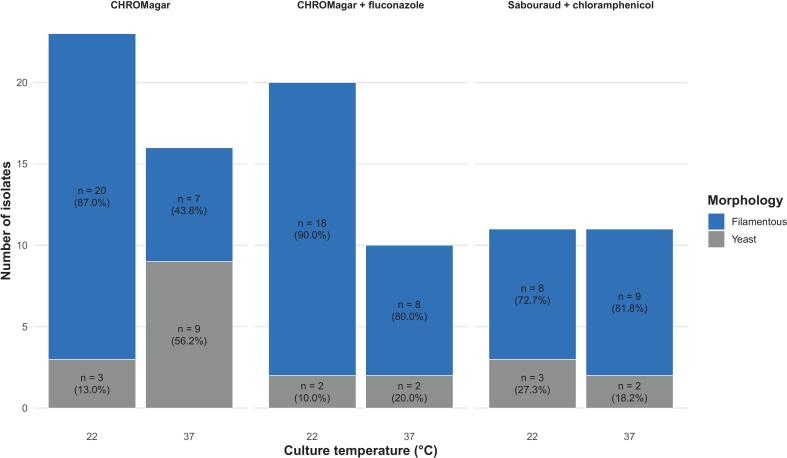
Distribution of fungal morphologies isolated on three different fungal culture media at two incubation temperatures.

Taken together, filamentous morphology predominated under all media and temperatures; however, a relative increase in the frequency of yeast forms versus molds was observed when culturing at 37 °C across all media compared to 22 °C. CHROMagar-based media supported the highest number of isolates, particularly filamentous forms, while SDA-C yielded a more balanced distribution between morphologies (Fig. 2). Among the 70 filamentous isolates, 87.1% (*n* = 61) were recovered from dolphins with a previous history of fungal infection, whereas 12.9% (*n* = 9) were obtained from individuals without prior infection. In contrast, among the 21 yeast isolates, 71.4% (*n* = 15) were recovered from dolphins without a history of infection and 28.6% (*n* = 6) from those with previous infection. Although yeasts appeared proportionally more frequent in dolphins without prior fungal infection compared with filamentous fungi, this difference did not reach statistical significance (Fisher's exact test, *p* = 0.103).

**Fig. 3:**
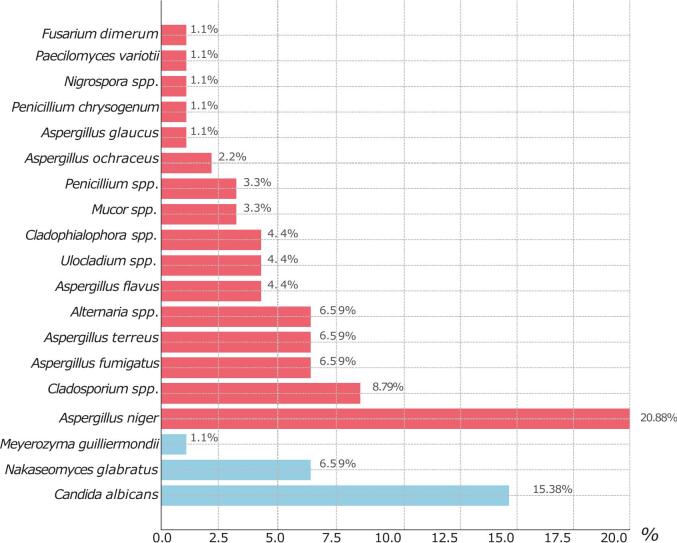
Distribution of the isolated fungi by genus or species from the respiratory tract of bottlenose dolphins

The yeast species isolated included *Candida albicans* (14 isolates, 15.38%), *Nakaseomyces glabratus* (former *Candida glabrata*) (6 isolates, 6.59%), and *Meyerozyma guilliermondii* (former *Candida guilliermondii*) (1 isolate, 1.10%) (Fig. 3).

Greater diversity and higher count were observed among the filamentous fungi. *Aspergillus* species were predominant, with *Aspergillus niger* being the most frequently isolated (19 isolates, 20.88%). Other *Aspergillus* species included *A. fumigatus* and *A. terreus* (6 isolates each, 6.59%) *A. flavus* (4 isolates, 4.40%), *A. ochraceus* (2 isolates, 2.20%), and *A. glaucus* (1 isolate, 1.10%) (Fig. 3). These two cryptic *Aspergillus* isolates were identified using both MALDI-TOF MS and Sanger sequencing of the β-tubulin and calmodulin genes, as detailed in the Methods section.

Other filamentous fungi included *Cladosporium* spp. (8 isolates, 8.79%), *Alternaria* spp. (6 isolates, 6.59%), *Ulocladium* spp. (4 isolates, 4.40%), *Cladophialophora* spp. (4 isolates, 4.40%), *Mucor* spp. (3 isolates, 3.30%), *Penicillium* spp. (3 isolates, 3.30%), *Penicillium chrysogenum* (1 isolate, 1.10%), *Fusarium dimerum* (1 isolate, 1.10%), *Nigrospora* spp. (1 isolate, 1.10%), and *Paecilomyces variotii* (1 isolate, 1.10%).

Membrane filtration was used to detect pathogenic fungi in aquarium water. However, no *Candida* spp., *Aspergillus* spp., *Mucorales* or *Fusarium* spp. were isolated. After incubation of the filters on SDA-C medium at 37 °C, no yeasts were observed but a single isolate of *Rhodotorula mucilaginosa*. This isolate was not found in any blow samples from the dolphins.

Regarding antifungal resistance, azole-resistance screening using VIPcheck plates was performed in 22 filamentous fungal isolates with recognized pathogenic potential, including 20 *Aspergillus* spp. isolates and 2 *Mucor* spp. isolates. The *Aspergillus* isolates screened comprised *A. niger, A. terreus, A. fumigatus, A. flavus*, and *A. ochraceus*. No growth was observed in the itraconazole- or voriconazole-containing wells among *Aspergillus* isolates. However, both *Mucor* spp. isolates grew in the presence of itraconazole, voriconazole, and posaconazole (from dolphins D1 and D7). Among *Aspergillus* isolates, one strain of *A. terreus* (from dolphin D3) and one strain of *A. niger* (from dolphin D9) showed growth in the posaconazole-containing well.

Twenty-one yeast isolates underwent susceptibility testing with YO10 plates including isavuconazole. Table 2 presents the minimum inhibitory concentration (MIC) ranges for nine antifungal agents tested against the *C. albicans*, *N. glabratus*, and *M. guilliermondii* isolates. Regarding triazole antifungals, all 14*C. albicans* isolates were fully susceptible to fluconazole, while 16.7% of *N. glabratus* isolates (1/6) were resistant (from dolphin D8). Resistance to itraconazole was detected in 50% of *C. albicans* isolates (7/14) (from dolphins D1 and D2), with the remaining 50% being fully susceptible. In *N. glabratus*, 16.7% of isolates (1/6) demonstrated resistance to itraconazole (dolphin D8), while 83.3% were susceptible. All *C. albicans* isolates (14/14) were susceptible to voriconazole, whereas in *N. glabratus*, 83.3% (5/6) were susceptible, and 16.7% (1/6) showed resistance (again, the isolate from dolphin D8). Similar results were obtained for posaconazole, where all *C. albicans* isolates were susceptible (14/14), and 83.3% of *N. glabratus* isolates (5/6) were also susceptible, with 16.7% (1/6) showing resistance (dolphin D8). For isavuconazole, isolates with MIC values exceeding two dilution steps above the modal MIC (0.015 mg/L) were considered non-wild type (non-wt) in the absence of established EUCAST epidemiological cutoff values (ECOFFs). All *C. albicans* isolates were classified as wild type (wt). In contrast, 16.7% of *N. glabratus* isolates (1/6) were non-wt for isavuconazole. The *N. glabratus* isolate in dolphin D8 which demonstrated cross-resistance to other azoles was non-isavuconazolewt.

**Table 2:** Summary of minimum inhibitory concentration (MIC) values for 9 antifungal agents in yeast isolates.

Species	AMB	FLC	ITC	VRC	CAS	ANF	MCF	POS	ISA
*Candida albicans* (*n* = 14)	0.25–0.5	0.5–2	0.03–0.125	0.008–0.03	<0.015–0.125	<0.015–0.03	<0.008–0.015	0.03–0.06	<0.008–0.03
*Nakaseomyces glabratus* (*n* = 6)	0.12–0.5	0.5–128	0.06–1	0.015–1	0.03–0.06	0.015–0.03	0.015–0.03	0.03–2	0.008–1
*Meyerozyma guilliermondii* (n = 1)	0.25	4	0.125	0.06	0.5	1	0.5	0.06	0.06

Note: AMB, amphotericin B; FLC, fluconazole; ITC, itraconazole; VRC, voriconazole; CAS, caspofungin; ANF, anidulafungin; MCF, micafungin; POS, posaconazole; ISA, isavuconazole. MIC values are expressed in mg/L.

Echinocandin susceptibility was uniformly high, with all *C. albicans* and *N. glabratus* isolates demonstrating susceptibility to caspofungin, micafungin, and anidulafungin (100%). No resistance to amphotericin B was observed across all isolates, regardless of species. Further information is represented in Supplementary table S3.

Both *C. albicans* strains showing itraconazole resistance were isolated from dolphins which had a history of long-term use of itraconazole as a therapeutic or prophylactic regimen, respectively. However, the *N. glabratus* strain showing cross-resistance to tested azoles was isolated from a dolphin, which had previously received therapy with itraconazole, terbinafine, and nystatin orally, but no other azoles.

## 4 Discussion

In this study, we performed a comprehensive culture-based characterization of the respiratory fungal community in common bottlenose dolphins under human care, integrating differential thermal incubation to prioritize thermotolerant taxa with potential pathogenic relevance and conducting antifungal susceptibility screening in clinically significant isolates. We identified 19 fungal taxa across the study population, revealing a varied fungal community dominated by filamentous fungi, particularly *Aspergillus* species, alongside clinically relevant yeasts such as *C. albicans* and *N. glabratus*. Fungal recovery was influenced by incubation temperature and culture medium. Antifungal susceptibility testing identified resistance to azoles in determined yeast and reduced susceptibility to azoles in selected filamentous fungal isolates, with potentially resistant *Candida*, *Aspergillus* and *Mucor* species, including patterns of multidrug resistance in some strains. Most resistant isolates were detected in dolphins with a history of antifungal exposure, raising concern about potential selective pressure in managed settings.

Fungal colonization in cetaceans remains poorly understood, with most of the available evidence limited to isolated case reports of infection by yeasts and filamentous fungi. There is scarce information in relation to microbiological procedures for fungal screening in these species [26]. Our group, in recent reviews, has noted that only a few studies to date have explored the composition, ecology, and profiles of antifungal resistance of the cetacean mycobiome on a species and habitat level [20], [27], [28]. This gap is particularly relevant given the zoonotic potential of many fungal species capable of infecting or colonizing marine mammals. To the best of our knowledge, no studies have comprehensively addressed the ecological occurrence of both environmental and pathogenic fungi yeasts and molds alike as well as incorporated antifungal susceptibility testing, an extremely valuable consideration in light of growing concern regarding resistance in fungal epidemiology.

On the one hand, although no studies have looked at filamentous fungi in captive cetaceans aside from single-case reports of severe infections, there have been some reports in the literature that included the isolation of human-associated yeasts like *C. albicans* and *C. tropicalis* from pool water and feces of healthy dolphins and one beluga whale housed in Florida [29]. Later investigations in Okinawa detected *Candida* spp. in exhaled breath of various captive cetaceans and *C. albicans* again being the most prevalent, with *C. tropicalis* and *N. glabratus* [19]. Environmental and staff samples in the study revealed shared genotypes of *Candida* spp. among dolphins, human beings, and in water, suggesting likely interspecies transmission and environmental shedding [19]. In our work, however, no pathogenic species were observed in the filtered pool water. Consistent with our findings, which identified azole-resistant isolates with preserved susceptibility to echinocandins and amphotericin B in yeasts, previous studies have also reported high rates of azole resistance [23], [24]. However, while prior antifungal exposure is a known risk factor, environmental selection pressures -such as the widespread use of agricultural azoles-may also contribute significantly to azole tolerance or resistance, even in treatment-naive animals. A substantial proportion of *C. albicans* and *C. tropicalis* isolates exhibited resistance or reduced susceptibility to fluconazole and itraconazole, with some *C. albicans* strains additionally resistant to 5-flucytosine [19].

Contrarily to our cohort, in which resistance in yeasts could partially be explained in some cases by prior selection by antifungal agent use, azole-resistant *Candida* spp. were isolated in Nagoya in 2019 from blowhole swabs of symptomatic and asymptomatic dolphins under human care, including itraconazole- and voriconazole-resistant isolates in animals never exposed to azoles [24]. Likewise, Shirakata et al. (2022) reported *C. albicans* isolates from dolphin respiratory breath in Enoshima that were equally resistant to fluconazole, itraconazole, and voriconazole, despite minimal prior history of antifungal therapy, although all were remained sensitive to amphotericin B and micafungin [19].

Curiously, while *C. tropicalis* was commonly reported in Asian facilities, it was not isolated in our study. Instead, we encountered *M. guilliermondii*, a species previously isolated from free-ranging bottlenose dolphins in the Gulf of Mexico [30].

Although filamentous fungi represented the predominant morphological group in our cohort, their role in respiratory colonization dynamics in cetaceans under human care remains largely unexplored, in contrast to the limited but more focused studies addressing *Candida* species. Many of the taxa identified are commonly detected in environmental air and aquatic systems; however, their recovery from respiratory samples under thermotolerant conditions supports their potential biological relevance, as environmental ubiquity does not exclude opportunistic colonization in mammals. Thermotolerance constitutes a key functional trait associated with pathogenic potential in warm-blooded hosts and may therefore help distinguish incidental environmental exposure from clinically meaningful taxa.

Current knowledge on filamentous fungi in cetaceans is largely restricted to isolated case reports of infection, predominantly involving *Aspergillus* spp., with *A. fumigatus* disproportionately represented compared to other less frequently described species [20], [31]. Importantly, species-level identification is often incomplete in published cases, and antifungal susceptibility data remain scarce. To date, only two studies have systematically addressed antifungal susceptibility in filamentous fungal isolates from cetaceans [32], [33].

In our study, we isolated several pathogenic *Aspergillus* species, with *A. niger* being the most frequently detected. Additionally, species not previously reported in cetaceans, such as *A. glaucus*, *A. ochraceus*, and *Fusarium dimerum*, were also identified. However, *A. fumigatus*, the species most associated with invasive aspergillosis in both veterinary and human medicine, was isolated only in one animal in our cohort. This may suggest that *A. fumigatus* acts more as an opportunistic pathogen than a regular colonizer in healthy animals, unlike *A. niger*, which appears more frequently in asymptomatic hosts. Nonetheless, other clinically relevant species such as *A. flavus* and *A. terreus* (both of which were detected) should not be underestimated, as they are also implicated in invasive disease [34] and may constitute part of the normal respiratory mycobiome. Additionally, in our exploratory screening, one isolate each of *A. niger* and *A. terreus* demonstrated screening indication of resistance with growth at elevated concentrations of posaconazole. This finding highlights the need for targeted surveillance of antifungal resistance in *Aspergillus* species colonizing cetaceans. Environmental drivers, particularly the widespread use of azole-based agricultural fungicides, have been linked to the emergence of resistant *A. fumigatus* strains carrying *cyp51A* mutations, notably the TR34/L98H allele. This mutation has also been reported in a free-ranging harbor porpoise in the Netherlands [32], reinforcing an environmental route of resistance selection in cetaceans with One Health impact. Moreover, other clinically relevant fungi were identified, including *Mucor* spp., which showed growth under high concentrations of posaconazole and itraconazole, and environmental dematiaceous molds such as *Cladophialophora* spp., among others.

Finally, beyond the ecological significance of these findings, our results underscore the importance of incorporating antifungal susceptibility testing and epidemiological surveillance cultures into veterinary practice for cetaceans under human care. This is particularly relevant given the high colonization rates observed and the potential for fungal infections to emerge under predisposing conditions, most notably during or following antibiotic therapy for bacterial infections. With the growing prevalence of azole resistance in this setting, understanding local fungal epidemiology is essential to guide empirical antifungal therapy, improve clinical outcomes, and reduce the risk of treatment failure and further resistance development. In this regard, the use of chromogenic media for presumptive fast identification and antifungal supplementation might provide a cost-effective tool for efficient surveillance and therapeutic guidance in this setting [26].

However, several limitations deserve mention. The sample size was restricted to ten dolphins from a single facility, which may limit the generalizability of the findings to other populations or environments, especially considering the apparent geographic differences with previous evidence. Consistent with this limitation, the species accumulation analysis suggested that although a substantial proportion of the cultivable fungal diversity was captured, the curve did not reach a clear asymptote, indicating that additional sampling could still reveal further fungal taxa. This pattern is consistent with heterogeneous fungal colonization among individuals, where rare taxa are detected only when sampling effort increases. Second, the cross-sectional and culture-dependent design does not allow discrimination between transient environmental exposure and stable respiratory colonization, and may underestimate total fungal diversity compared to high-throughput sequencing approaches. Although we explored antifungal susceptibility, only a first screening was performed for molds, and resistance mechanisms were not further characterized by molecular methods. A high proportion of animals in this cohort had a history of suspected or confirmed fungal infection and prior antifungal exposure. However, veterinary records indicate that antifungal therapy was frequently initiated empirically and that previous isolates were often not identified beyond the genus level. Consequently, direct comparison between historical infections and the fungi recovered in the present study was not feasible, and the contribution of prior antifungal exposure to the resistance patterns observed here should be interpreted with caution in the context of a cross-sectional approach. Additionally, despite we did not find pathogenic species in water samples, veterinary or human staff were not sampled, who could be linked to interspecific transmission.

## 5 Conclusions

This study offers a comprehensive culture-based characterization of the respiratory fungal community and antifungal resistance patterns in bottlenose dolphins under human care. Our findings reveal a diverse fungal community dominated by filamentous fungi, including pathogenic *Aspergillus* species, and clinically relevant yeasts with notable selection of azole resistance profiles. A total of 19 fungal taxa were identified, with a predominance of *Aspergillus* spp. and the recovery of yeasts such as *Candida albicans* and *Nakaseomyces glabratus*, alongside preserved susceptibility to echinocandins and amphotericin B. The detection of resistant strains, including multidrug-resistant strains, underscores the importance of routine mycological surveillance and antifungal susceptibility testing in cetacean veterinary care. These results highlight the potential of dolphins as sentinels for environmental fungal resistance and reinforce the need for a One Health approach to fungal ecology and emerging threats.

## Authors contribution

All authors contributed to the study conception and design. VGB, ACRG, JMPR and MDCN performed microbiological analysis. TA, CRS, and MV carried out the sampling procedure. VGB reviewed the evidence, analyzed the data, constructed tables and figures, and wrote the first version of the article. MDCN aided in literature research, construction of tables and figures, and language revision. BAH and IRM supervised the work, interpreted the data and did the literature review. All authors commented on previous versions of the manuscript. All authors read and approved the final manuscript.

## CRediT authorship contribution statement

**Victor Garcia-Bustos:** Writing – review & editing, Writing – original draft, Visualization, Validation, Software, Resources, Project administration, Methodology, Investigation, Funding acquisition, Formal analysis, Data curation, Conceptualization. **Alba Cecilia Ruiz-Gaitán:** Writing – review & editing, Validation, Methodology, Formal analysis. **Begoña Acosta-Hernández:** Writing – original draft, Validation, Supervision, Resources, Project administration, Methodology, Investigation, Data curation, Conceptualization. **Teresa Álvaro:** Writing – review & editing, Methodology, Investigation, Data curation. **Carlos Rojo-Solís:** Writing – review & editing, Methodology, Investigation, Data curation. **Mónica Valls:** Writing – review & editing, Methodology, Investigation, Data curation. **José Manuel Pérez-Royo:** Writing – review & editing, Methodology, Formal analysis. **Inmaculada Rosario Medina:** Writing – review & editing, Validation, Supervision, Resources, Project administration, Methodology, Investigation, Data curation, Conceptualization.

## Funding

This work was funded through the “Dr. Juan Peset Aleixandre” grant from the Valencia City Council (Ajuntament de València), awarded by the Valencian Medical Institute. This study was also supported by the 10.13039/501100004587Instituto de Salud Carlos III (ISCIII) through Juan Rodés and Miguel Servet contracts (JR24/00020, CP25/00054) awarded to the first and corresponding author, as well as by project PI25/00654, co-funded by the 10.13039/501100000780European Union.

## Declaration of competing interest

The authors declare that they have no known competing financial interests or personal relationships that could have appeared to influence the work reported in this paper.

## Data Availability

Data will be made available on request.

## Appendix A Supplementary data

Supplementary material: fungal diversity, culture characteristics, and antifungal susceptibility profiles of isolates recovered from dolphins.
