# Supplementary material for: Culture-based characterization of the respiratory mycobiota and antifungal resistance in bottlenose dolphins under human care
Source: One Health. 2026 May 16;22:101439. doi: 10.1016/j.onehlt.2026.101439 (PMC13213738; doi:10.1016/j.onehlt.2026.101439)

Supplementary material

Table S1. Number of fungal isolates and species recovered from each dolphin included in the study. Species counts correspond to the number of distinct taxa detected per dolphin.

| Dolphin ID | No. of isolates | No. of species | Yeast species (n) | Filamentous species (n) |
| --- | --- | --- | --- | --- |
| D1 | 9 | 5 | 2 | 3 |
| D2 | 8 | 4 | 2 | 2 |
| D3 | 10 | 8 | 0 | 8 |
| D4 | 7 | 5 | 0 | 5 |
| D5 | 11 | 8 | 1 | 7 |
| D6 | 8 | 3 | 0 | 3 |
| D7 | 17 | 11 | 1 | 10 |
| D8 | 7 | 2 | 1 | 1 |
| D9 | 10 | 4 | 1 | 3 |
| D10 | 4 | 4 | 1 | 3 |

Figure S1: Number of fungal species per dolphin


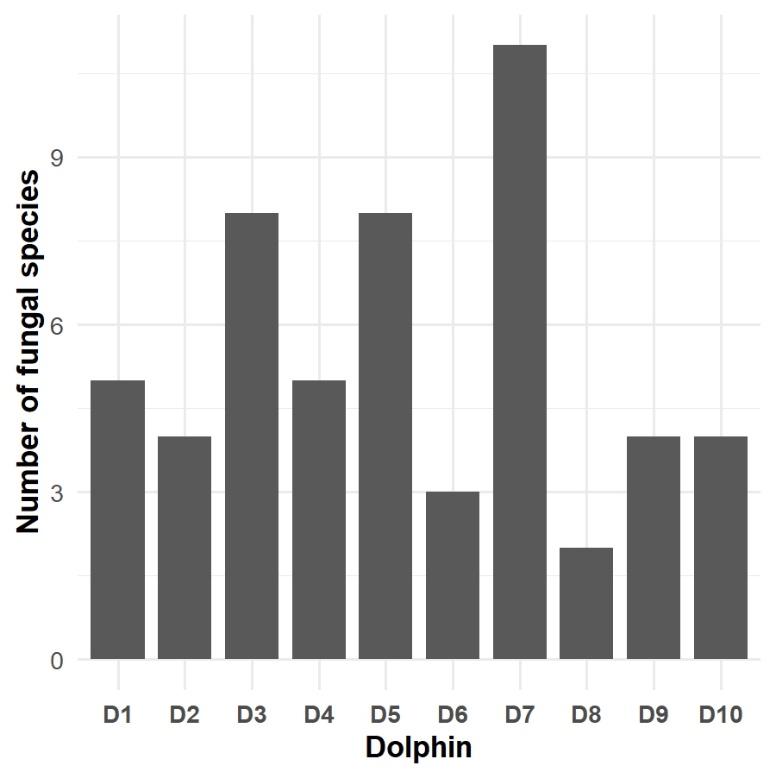


Figure S2. Number of fungal species according to culture temperature


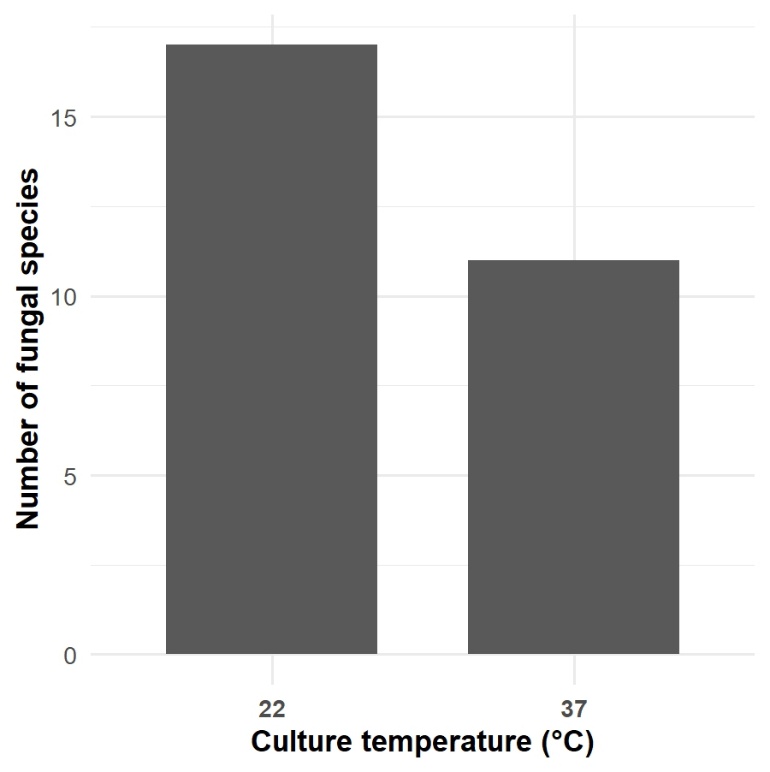


Table S2. Fungal species recovered from each dolphin, including number of isolates, incubation temperature, culture medium, and morphological classification. CROM: CHROMagar *Candida*; CHROMFLUCO32: CHROMagar *Candida* with fluconazole 32 mg/L. Fil: filamentous; Tª: temperature; SABCLOR: Sabouraud chloramphenicol

| **Dolphin** | **Species** | **N** | **Tª** | **Media** | **Morphology** |
| --- | --- | --- | --- | --- | --- |
| D1 | *Aspergillus flavus* | 1 | 37 | CROMFLUCO32 | Fil |
| D1 | *Aspergillus niger* | 1 | 22 | CROM | Fil |
| D1 | *Candida albicans* | 2 | 22, 37 | CROM | Yeast |
| D1 | *Nakaseomyces glabratus* | 4 | 22, 37 | CROM, CROMFLUCO32 | Yeast |
| D1 | *Mucor spp.* | 1 | 37 | CROM | Fil |
| D2 | *Aspergillus flavus* | 1 | 37 | CROMFLUCO32 | Fil |
| D2 | *Aspergillus ochraceus* | 1 | 22 | SABCLOR | Fil |
| D2 | *Candida albicans* | 5 | 22, 37 | CROM, SABCLOR | Yeast |
| D2 | *Meyerozyma guilliermondii* | 1 | 22 | CROMFLUCO32 | Yeast |
| D3 | *Aspergillus flavus* | 1 | 37 | CROMFLUCO32 | Fil |
| D3 | *Aspergillus fumigatus* | 1 | 22 | SABCLOR | Fil |
| D3 | *Aspergillus glaucus* | 1 | 22 | CROMFLUCO32 | Fil |
| D3 | *Aspergillus ochraceus* | 1 | 37 | CROMFLUCO32 | Fil |
| D3 | *Aspergillus terreus* | 1 | 37 | SABCLOR | Fil |
| D3 | *Cladosporium spp.* | 3 | 22 | CROM, CROMFLUCO32 | Fil |
| D3 | *Nigrospora spp.* | 1 | 22 | CROMFLUCO32 | Fil |
| D3 | *Ulocladium spp.* | 1 | 22 | CROM | Fil |
| D4 | *Alternaria spp.* | 2 | 22, 37 | CROM, SABCLOR | Fil |
| D4 | *Aspergillus flavus* | 1 | 37 | SABCLOR | Fil |
| D4 | *Cladophialophora spp.* | 1 | 22 | CROM | Fil |
| D4 | *Cladosporium spp.* | 1 | 37 | CROMFLUCO32 | Fil |
| D4 | *Penicillium spp.* | 2 | 22 | CROM | Fil |
| D5 | *Alternaria spp.* | 2 | 22 | CROM, CROMFLUCO32 | Fil |
| D5 | *Aspergillus niger* | 1 | 22 | CROMFLUCO32 | Fil |
| D5 | *Candida albicans* | 2 | 22, 37 | SABCLOR | Yeast |
| D5 | *Cladophialophora spp.* | 1 | 22 | CROMFLUCO32 | Fil |
| D5 | *Cladosporium spp.* | 1 | 22 | CROM | Fil |
| D5 | *Mucor spp.* | 1 | 22 | SABCLOR | Fil |
| D5 | *Paecilomyces variotii* | 1 | 22 | SABCLOR | Fil |
| D5 | *Ulocladium spp.* | 2 | 22 | CROMFLUCO32, SABCLOR | Fil |
| D6 | *Aspergillus fumigatus* | 2 | 37 | CROMFLUCO32, SABCLOR | Fil |
| D6 | *Aspergillus niger* | 5 | 22, 37 | CROM, CROMFLUCO32, SABCLOR | Fil |
| D6 | *Penicillium chrysogenum* | 1 | 37 | CROM | Fil |
| D7 | *Alternaria spp.* | 2 | 22 | CROM, CROMFLUCO32 | Fil |
| D7 | *Aspergillus fumigatus* | 1 | 22 | CROM | Fil |
| D7 | *Aspergillus niger* | 5 | 22, 37 | CROM, CROMFLUCO32, SABCLOR | Fil |
| D7 | *Aspergillus terreus* | 1 | 37 | CROM | Fil |
| D7 | *Candida albicans* | 1 | 37 | CROM | Yeast |
| D7 | *Cladophialophora spp.* | 1 | 22 | CROMFLUCO32 | Fil |
| D7 | *Cladosporium spp.* | 2 | 22 | CROM, CROMFLUCO32 | Fil |
| D7 | *Fusarium dimerum* | 1 | 22 | CROM | Fil |
| D7 | *Mucor spp.* | 1 | 22 | SABCLOR | Fil |
| D7 | *Penicillium spp.* | 1 | 22 | CROMFLUCO32 | Fil |
| D7 | *Ulocladium spp.* | 1 | 22 | CROM | Fil |
| D8 | *Aspergillus niger* | 5 | 22, 37 | CROM, CROMFLUCO32, SABCLOR | Fil |
| D8 | *Nakaseomyces glabratus* | 2 | 37 | CROM, CROMFLUCO32 | Yeast |
| D9 | *Aspergillus fumigatus* | 2 | 22, 37 | CROMFLUCO32, SABCLOR | Fil |
| D9 | *Aspergillus niger* | 1 | 22 | CROMFLUCO32 | Fil |
| D9 | *Aspergillus terreus* | 4 | 22, 37 | CROM, CROMFLUCO32, SABCLOR | Fil |
| D9 | *Candida albicans* | 3 | 22, 37 | CROM, SABCLOR | Yeast |
| D10 | *Aspergillus niger* | 1 | 22 | CROM | Fil |
| D10 | *Candida albicans* | 1 | 37 | CROM | Yeast |
| D10 | *Cladophialophora spp.* | 1 | 22 | CROM | Fil |
| D10 | *Cladosporium spp.* | 1 | 22 | CROM | Fil |

Table S3: Detail of minimum inhibitory concentration (MIC) values for 9 antifungal agents in yeast isolates

| Animal | Isolate | Species | AMB | FLC | ITC | VRC | CAS | ANF | MCF | POS | ISA |
| --- | --- | --- | --- | --- | --- | --- | --- | --- | --- | --- | --- |
| D1 | A.0823.01.01 | *Candida albicans* | 0.5 | 2 | 0.125* | 0.03 | 0.125 | 0.03 | 0.015 | 0.03 | 0.03 |
| D1 | A.0823.01.06 | *Candida albicans* | 0.5 | 2 | 0.125* | 0.03 | 0.125 | 0.03 | 0.015 | 0.03 | 0.03 |
| D2 | B.0823.01.01 | *Candida albicans* | 0.5 | 1 | 0.125* | 0.03 | 0.03 | 0.03 | 0.015 | 0.06 | 0.015 |
| D2 | B.0823.01.02 | *Candida albicans* | 0.5 | 1 | 0.125* | 0.03 | 0.03 | 0.03 | 0.015 | 0.06 | 0.015 |
| D2 | B.0823.01.03 | *Candida albicans* | 0.5 | 1 | 0.125* | 0.03 | 0.03 | 0.03 | 0.015 | 0.06 | 0.015 |
| D2 | B.0823.01.06 | *Candida albicans* | 0.5 | 1 | 0.125* | 0.03 | 0.03 | 0.03 | 0.015 | 0.06 | 0.015 |
| D2 | B.0823.01.09 | *Candida albicans* | 0.5 | 1 | 0.125* | 0.03 | 0.03 | 0.03 | 0.015 | 0.06 | 0.015 |
| D5 | KU.0923.01.1 | *Candida albicans* | 0.5 | 0.5 | 0.06 | 0.015 | 0.03 | 0.015 | 0.015 | 0.03 | 0.015 |
| D5 | KU.0923.01.2 | *Candida albicans* | 0.5 | 0.5 | 0.06 | 0.015 | 0.03 | 0.015 | 0.015 | 0.03 | 0.015 |
| D7 | LE.0923.01.4 | *Candida albicans* | 0.25 | 0.5 | 0.03 | 0.008 | 0.03 | 0.015 | 0.01 | 0.03 | 0.008 |
| D9 | N.0823.01.01 | *Candida albicans* | 0.5 | 2 | 0.03 | 0.015 | 0.015 | 0.015 | 0.015 | 0.03 | 0.015 |
| D10 | N.0823.01.04 | *Candida albicans* | 0.5 | 2 | 0.03 | 0.015 | 0.015 | 0.015 | 0.015 | 0.03 | 0.015 |
| D11 | N.0823.01.12 | *Candida albicans* | 0.5 | 2 | 0.03 | 0.015 | 0.015 | 0.015 | 0.015 | 0.03 | 0.015 |
| D10 | U.0923.01.1 | *Candida albicans* | 0.25 | 0.5 | 0.03 | 0.015 | 0.06 | <0,015 | <0.008 | 0.03 | <0,008 |
| D1 | A.0823.01.03 | *Nakaseomyces glabratus* | 0.5 | 0.5 | 0.06 | 0.015 | 0.03 | 0.03 | 0.015 | 0.03 | 0.008 |
| D1 | A.0823.01.05 | *Nakaseomyces glabratus* | 0.5 | 0.5 | 0.06 | 0.015 | 0.03 | 0.03 | 0.015 | 0.03 | 0.008 |
| D1 | A.0823.01.07 | *Nakaseomyces glabratus* | 0.5 | 0.5 | 0.06 | 0.015 | 0.03 | 0.03 | 0.015 | 0.03 | 0.008 |
| D1 | A.0823.01.10 | *Nakaseomyces glabratus* | 0.5 | 0.5 | 0.06 | 0.015 | 0.03 | 0.03 | 0.015 | 0.03 | 0.008 |
| D8 | NA.0923.01.3 | *Nakaseomyces glabratus* | 0.12 | 2 | 0.06 | 0.03 | 0.06 | 0.015 | 0.015 | 0.125 | 0.008 |
| D9 | NA.0923.01.7 | *Nakaseomyces glabratus* | 0.12 | 128* | 1* | 1* | 0.06 | 0.03 | 0.03 | 2* | 1 |
| D2 | B.0823.01.04 | *Meyerozyma guilliermondii* | 0.25 | 4 | 0.125 | 0.06 | 0.5 | 1 | 0.5 | 0.06 | 0.06 |

Note: AMB, amphotericin B; FLC, fluconazole; ITC, itraconazole; VRC, voriconazole; CAS, caspofungin; ANF, anidulafungin; MCF, micafungin; POS, posaconazole; ISA, isavuconazole. MIC values are expressed in mg/L. Asterisk: values considering resistant following the clinical breakpoints for fungi v. 12.0 from the European Committee on Antimicrobial Susceptibility Testing (EUCAST).

Figure S3: Species accumulation curve showing the cumulative number of fungal species detected as a function of the number of dolphins sampled. The curve represents the mean species richness estimated from random permutations of sampling order, and the shaded area indicates the standard deviation around the mean.


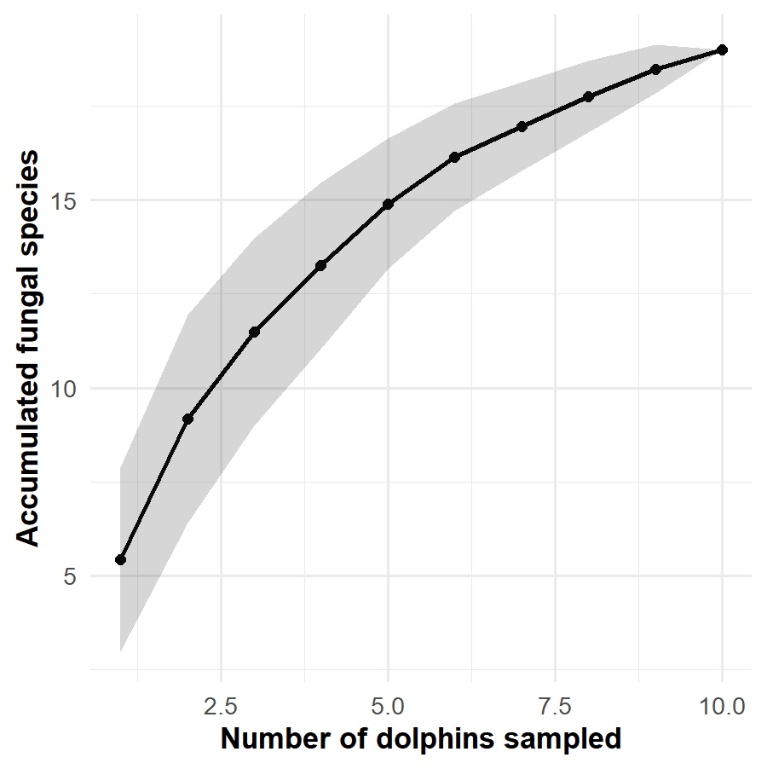

Supplement: Supplementary file 1 — Supplementary material: fungal diversity, culture characteristics, and antifungal susceptibility profiles of isolates recovered from dolphins. [file mmc1.docx]
